# Folinic Acid Prophylaxis and Dose Adjustments Enable Safe Treatment with Pemetrexed in Patients with Renal Impairment

**DOI:** 10.1002/cpt.3735

**Published:** 2025-05-27

**Authors:** Nikki de Rouw, Leila‐Sophie Otten, Mart P. Kicken, Berber Piet, Bonne Biesma, Bianca van Veggel, Christi M.J. Steendam, Ben van den Borne, Lizza E.L. Hendriks, Sander Croes, Anne‐Marie C. Dingemans, Daphne W. Dumoulin, Ron H.J. Mathijssen, Jacobus A. Burgers, Alwin D.R. Huitema, Hieronymus J. Derijks, Maarten J. Deenen, Michel M. van den Heuvel, Rob ter Heine

**Affiliations:** ^1^ Department of Clinical Pharmacy Amphia Hospital Breda The Netherlands; ^2^ Department of Pharmacy, Pharmacology & Toxicology Research Institute for Medical Innovation, Radboudumc Nijmegen The Netherlands; ^3^ Department of Clinical Pharmacy Catharina Hospital Eindhoven The Netherlands; ^4^ Department of Pulmonary Diseases Research Institute for Medical Innovation, Radboudumc Nijmegen The Netherlands; ^5^ Department of Pulmonary Diseases Jeroen Bosch Hospital's Hertogenbosch The Netherlands; ^6^ Department of Pulmonary Diseases Catharina Hospital Eindhoven The Netherlands; ^7^ Department of Pulmonary Diseases GROW‐School for Oncology and Reproduction, Maastricht University Medical Center Maastricht The Netherlands; ^8^ Department of Clinical Pharmacy & Toxicology CARIM‐School for Cardiovascular Disease, Maastricht University Medical Centre Maastricht The Netherlands; ^9^ Department of Respiratory Medicine Erasmus MC Cancer Institute, University Medical Center Rotterdam the Netherlands; ^10^ Department of Medical Oncology Erasmus MC Cancer Institute Rotterdam The Netherlands; ^11^ Department of Thoracic Oncology Antoni Van Leeuwenhoek‐The Netherlands Cancer Institute Amsterdam The Netherlands; ^12^ Department of Pharmacy & Pharmacology Antoni Van Leeuwenhoek‐The Netherlands Cancer Institute Amsterdam The Netherlands; ^13^ Department of Clinical Pharmacy Utrecht University Medical Center, Utrecht University Utrecht The Netherlands; ^14^ Department of Pharmacology Princess Máxima Center for Pediatric Oncology Utrecht The Netherlands; ^15^ Department of Pharmacy Jeroen Bosch Hospital S‐Hertogenbosch The Netherlands; ^16^ Department of Clinical Pharmacy and Toxicology Leiden University Medical Center Leiden The Netherlands

## Abstract

Pemetrexed is a cornerstone in chemo(immunotherapy) of non‐small cell lung cancer and mesothelioma; however, it is contraindicated in patients with renal impairment due to severe toxicity concerns. Therefore, a large proportion of patients is withheld from effective chemo(immunotherapy). We performed an intra‐patient 3 + 3 dose escalation renal impairment study (eGFR < 45 mL/min). The pemetrexed dose was calculated based on renal function to reach a target AUC, and patients received oral folinic acid prophylaxis 45 mg four times daily on Days 2–15 of each cycle. Endpoints included safety (incidence of hematological and non‐hematological toxicity, treatment delays) and pharmacokinetics in line with regulatory guidance for renal impairment trials. Six patients with an estimated glomerular filtration rate (eGFR) between 26 and 41 mL/min were included. All patients were successfully escalated to the full dose. Adverse event patterns and pharmacokinetics were comparable to those in patients with normal renal function. Grade I/II anemia occurred in five patients (already present at baseline). One occurrence of grade IV neutropenia was observed, which resolved without intervention. Moreover, in three patients, a 1‐week treatment delay occurred. Treatment resulted in a response in four patients (*n* = 1 complete response, *n* = 2 partial response, *n* = 1 stable disease). Pemetrexed can be safely administered in patients with impaired renal function when the dose is calculated based on renal function and folinic acid prophylaxis is administered, thereby enabling an effective treatment modality for patients that, thus far, could not be treated.

**Study Highlights**

**WHAT IS THE CURRENT KNOWLEDGE ON THE TOPIC?**

Pemetrexed remains to be a cornerstone in treating NSCLC and mesothelioma, but impaired renal function is a contraindication. Without chemotherapy background, immune checkpoint inhibition is not effective in NSCLC patients with low PDL1 expression. Previous attempts to study dosing of pemetrexed in renal impairment were unsuccessful. A safe treatment strategy with pemetrexed for this vulnerable patient group is thus lacking.

**WHAT QUESTION DID THIS STUDY ADDRESS?**

Can we develop a safe treatment strategy to use pemetrexed in patients with renal impairment using folinic acid prophylaxis?

**WHAT DOES THIS STUDY ADD TO OUR KNOWLEDGE?**

We showed that pemetrexed dosing based on renal function combined with oral folinic acid prophylaxis enables safe treatment and therapeutic exposure in patients with renal impairment who cannot otherwise receive effective chemoimminutherapy treatment. This can be directly impemented in the clinic.

**HOW MIGHT THIS CHANGE CLINICAL PHARMACOLOGY OR TRANSLATIONAL SCIENCE?**

This study shows how combining pharmacological knowledge from preclinical‐, modeling‐, and clinical studies can lead to enablement of treatment for special patients populations.

Pemetrexed is the chemotherapeutic mainstay in the treatment of advanced non‐squamous non‐small cell lung cancer (NSCLC). For this indication, a chemotherapy background regimen consisting of pemetrexed with a platinum drug is a prerequisite for effective immune checkpoint inhibitor treatment in patients with low programmed death ligand 1 (PD‐L1) expression,^1^ accounting for a third to a half of all advanced NSCLC patients. Renal impairment is a common comorbidity in these patients, with an incidence of up to 30%.^2^ Due to fatal toxicities in a renal impairment study of pemetrexed during the clinical development of this drug by the license holder, renal impairment (defined as a creatinine clearance < 45 mL/min) is a contraindication.^3^, ^4^ Consequently, patients with renal impairment are withheld both effective chemotherapy and immunotherapy, showing the urgent need to develop a safe dosing regimen for pemetrexed in this vulnerable patient population.

By means of robust translational pharmacokinetic/pharmacodynamic modeling, we previously unraveled the pemetrexed pharmacokinetics‐toxicity mechanism^5^ and showed that (1) the toxicity of pemetrexed is driven by a time above a threshold concentration in plasma and (2) that a therapeutic pemetrexed dose cannot be safely administered in patients without toxicity prophylaxis. Folinic acid can antagonize the toxic effects of pemetrexed and is already considered in the label to treat severe pemetrexed toxicity.^6^ Folinic acid differs from the standard prophylaxis with folic acid, which is standard care for patients receiving pemetrexed. Folinic acid is the reduced form of folate and is able to bypass the blockage of the downstream enzyme dihydrofolatereductase (DHFR) in the folate cycle by pemetrexed, especially in healthy cells.^6^ No study has, thus far, been performed to investigate the potential of folinic acid prophylaxis to prevent pemetrexed‐induced toxicity in patients with renal impairment. The aim of our clinical study was, therefore, to develop a safe treatment strategy to use pemetrexed in patients with renal impairment using folinic acid prophylaxis.

## MATERIALS AND METHODS

### Study population

All patients with an indication for pemetrexed‐containing therapy, estimated glomerular filtration rate (eGFR) or creatinine clearance < 45 mL/min, and willing to provide written informed consent were eligible for inclusion. Concomitant use of non‐steroidal anti‐inflammatory drugs (NSAIDs) was not permitted during treatment with pemetrexed as per local protocols.

### Study design

The presented IMPROVE‐I was a sub‐study from the IMPROVE‐study (ZonMW grant number 848016010) that aimed at optimizing pemetrexed treatment. Whereas the IMPROVE‐I was a study for patients with renal impairment, the IMPROVE‐II was a randomized study that investigated the feasibility of individualized versus standard dosing in patients with normal renal function (clinicaltrials.gov NCT03655821).

We performed a renal impairment study compliant with EMA and FDA guidance for clinical studies in renal impairment,^7^, ^8^ to obtain a practice‐changing level of evidence. The main endpoints for this study were safety and pharmacokinetics, as these guidelines state that a dosing recommendation for impaired renal function should aim at the majority of patients being in the target range based on the therapeutic dose in the pivotal trial population (thus patients with normal renal function). We used a 3 + 3 intraindividual dose escalation design, commonly used for oncology trials to safely escalate the dose of cytotoxic drugs. The toxicity was graded according to the Common Terminology Criteria for Adverse Events (CTCAE) v5. A DLT was defined as a grade IV or higher hematological toxicity or any grade III or higher non‐hematological toxicity. The study was performed in Radboudumc Nijmegen, Catharina Hospital Eindhoven, and the Jeroen Bosch Hospital in Den Bosch, the Netherlands (Clinicaltrials.gov identifier is NCT03656549) and was performed according to the Declaration of Helsinki. The study was approved by the Medical Ethics Committee Oost‐Nederland in Nijmegen, the Netherlands, under registration number NL65481.091.18.

The study endpoints consisted of the fraction of patients safely treated with the full dose (see details below) and the area under the concentration‐time curve (AUC) of pemetrexed. Furthermore, the occurrence of dose‐limiting toxicities (DLT) and toxicity‐related treatment delays or discontinuations were obtained, as well as data on treatment response, as reported by the treating physician. The study was successful if all patients received the full dose with a maximum of 1 DLT in total and if the 90% confidence interval of the geometric mean of the pemetrexed AUC was within 164 mg*h/L ± 25%, previously proposed as a proxy for effective and safe exposure^7^, ^8^ and exposure that is associated with the approved dose in patients with adequate renal function.^9^

As treatment with pemetrexed in patients with impaired renal function is associated with severe myelosuppression in the absence of folinic acid,^3^, ^5^ inclusion was divided into two cohorts of 3 + 3.^10^ The first three patients were required to have a renal function between 30 and 45 mL/min (Cohort 1); for the remaining patients, any renal function < 45 mL/min was acceptable (Cohort 2). In Cohort 1 (*n* = 3), a subsequent patient was allowed to start one dose level below the maximum reached dose level of the previous patient at that timepoint. In Cohort 2 (*n* = 3), all patients were allowed to start at full dose (see “treatment”) if all patients in Cohort 1 reached the full dose and ≤ 1 DLT occurred.

### Treatment

In this study, pemetrexed dose is based on renal function (Chronic Kidney Disease Epidemiology Collaboration, CKD‐EPI equation), and calculated to reach a target AUC as described above. The pemetrexed dose for each patient was calculated according to **Eq**. **1** at each cycle (based on a validated population pharmacokinetic model^11^) to reach a target AUC of 164 mg*h/L.

(1) $$100\%\text{Dose}=109\times {\left(\frac{\text{weight}}{70}\right)}^{0.75}+561\times \left(\frac{\text{eGFR}}{70}\right)$$

This equation includes total body weight in kilograms and estimated glomerular filtration rate (eGFR) in mL/min corrected for individual BSA. Doses were rounded to the nearest 5 mg. Dose escalation steps were 10–33–66–100% of the dose for Cohort 1, escalated per cycle. The dose of 100% was defined as the dose that would result in an AUC of 164 mg*h/L.

Patients received standard vitamin supplementation (folic acid and vitamin B12) and dexamethasone according to the pemetrexed drug label. Oral folinic acid prophylaxis was initiated 24 hours after administration of pemetrexed in a dose of 45 mg four times daily on Days 2–15 of each 21‐day cycle. In case of ≥ 2 DLT in the two *n* = 3 cohorts, use of prophylactic granulocyte‐colony stimulating factor (G‐CSF) per protocol was permitted in an expansion cohort.

The rationale of the folinic acid strategy is based on several considerations. Comparable oral doses of 30‐45 mg four times daily are used in preventing toxicity from methotrexate, a more potent inhibitor of DHFR.^12^ The 24 hours' time interval ensures an efficacy window in malignant cells.^12^, ^13^, ^14^ Our previous validated pharmacokinetic/pharmacodynamic analysis in patients with adequate and impaired renal function using a neutropenia model for pemetrexed showed that 95% of patients in the lowest renal function group (eGFR of 5 mL/min) have pemetrexed concentrations below the established toxicity threshold after 10.6 days. Therefore, we postulate that 14 days of treatment with folinic acid is sufficient to prevent haematotoxicity.^5^

### Safety and laboratory measurements

Hematological laboratory measurements were performed according to the drug label and expected nadir in the dose escalation cycles. Treatment delay was allowed in case of grade III/IV toxicity; dose reductions were not permitted. Treatment with pemetrexed was discontinued if a patient experienced a DLT that did not resolve without intervention within 1 week. In case of persistent grade IV hematological toxicity, intravenous administration of 100 mg/m^2^ folinic acid every 6 hours until toxicity resolved was permitted. The AUC of pemetrexed was estimated using a validated limited sampling strategy.^15^

### Statistical analysis

The incidence of toxicity, treatment delays, and discontinuation were reported descriptively. To compare pharmacokinetics between patients with impaired and normal renal function, the 90% confidence interval of the geometric mean AUC was calculated and compared with the AUC and 90% confidence interval observed in patients with adequate renal function treated with pemetrexed dosed according to the drug label, from the standard of care arm (*n* = 44) of the previously published IMPROVE‐II trial.^16^

## RESULTS

### Patient characteristics

Eight patients were enrolled, of whom six were evaluable (**Table** **1**). Two patients dropped out due to rapid disease progression before the first possibility for pharmacokinetic evaluation. The line of treatment ranged from first‐ to third‐line treatment. Baseline absolute eGFR varied between 26 and 41 mL/min. At baseline, grade I/II anemia was present in five out of six patients. Four out of six patients received chemotherapy in combination with immunotherapy. NSAIDs were not used by any of the patients, three patients used a proton pump inhibitor (PPI). None of the patients used G‐CSF during treatment. The predefined expansion G‐CSF cohort was not required. Therefore, folinic acid prophylaxis alone was thus considered sufficient to prevent hematological toxicity.

**Table 1:** Overview of treatment and outcomes of all included patients at the start of treatment

	Cohort 1			Cohort 2			
Patient no	1	2	3	4	5	6	Group
*Baseline measurements*							
Sex (m/f)	f	m	m	m	f	m	
Age (years)	68	78	64	71	74	75	72 (64–78)
Weight (kg)	58	84	89	89	53	74	74 (53–89)
BSA (kg/m^2^)	1.68	2.03	2.13	2.06	1.53	1.95	1.9 (1.5–2.1)
eGFR (mL/min)	34	39	38	26	41	32	35 (26–41)
Hemoglobin (mmol/L)	7.3	7.9	9.2	6.9	6.4	5.3	7.2 (5.3–9.2)
Platelet count (×10^9^/L)	346	133	165	209	312	350	253 (133–350)
Red blood cell count (×10^9^/L)	3.69	4.01	4.94	3.88	4.24	3.00	3.96 (3.00–4.94)
White blood cell count (×10^9^/L)	8.0	7.1	11.4	6.8	10.6	9.0	8.8 (6.8–11.4)
Neutrophils (×10^9^/L)	6.70	4.46	nd	4.2	nd	6.2	5.4 (4.2–6.7)
NSCLC, stage	Stage IV	Stage IV	Stage IV	Stage IV	Stage III	Stage IV	
Line of systemic treatment	2	3	2	1	1	1	
Previous treatment	Cisplatin + etoposide	Ipilimumab + nivolumab cisplatin + pemetrexed	Pembrolizumab	–	–	–	
Combination therapy	Carboplatin Pembrolizumab	Carboplatin Pembrolizumab	Carboplatin	Carboplatin Pembrolizumab	Carboplatin	Carboplatin Pembrolizumab	–
Use of relevant co‐medication	–	–	PPI	PPI	–	PPI	
Number of cycles within study (*n*)	4	4	4	4	4	4	4
Dose escalation scheme	10–33–66‐100%	10–33–66‐100%	66–100%	100%	100%	100%	
Dose received (mg)	10%: 35	10%: 50	66%: 320	100%: 335	100%: 385	100%: 390	‐
	33%: 120	33%: 140	100%: 580	100%: 335	100%: 380	100%: 325	
	66%: 240	66%: 310	100%: 520	100%: 325	100%: 400	100%: 350	
	100%: 320	100%: 480	100%: 620	100%: 335	100%: 390	100%: 375	
*Pharmacokinetics* a							
V1 (L)	6.02	7.10	5.97	5.57	4.69	7.32	6.04 (5.77–6.58)
V2 (L)	7.47	7.77	6.80	6.35	5.45	8.47	6.98 (6.58–7.62)
V3 (L)	1.16	1.17	1.08	0.85	0.82	1.33	1.05 (0.95–1.17)
Cl (L/h)	2.35	2.49	3.03	1.90	2.48	2.23	2.39 (2.29–2.48)
AUC at 100% dose (mg*h/L)	136	192	191	176	155	175	170 (163–184)
*Grade III/IV hematotoxicity at nadir (n, cycle no)*							
Decreased neutrophil count	–	–	–	–	–	3 (c1, c2 c3)	3
Anemia	–	–	–	–	–	2 (c3, c4)	2
Decreased platelet count	–	1 (c4)	2 (c2, c4)	–	–	1 (c4)	3
*Grade III/IV hematotoxicity at day 21–28 (n, cycle no)*							
Decreased neutrophil count	–	–	–	–	–	3 (c1, c2 c3)	3
Anemia	–	–	–	–	–	2 (c3, c4)	2
Decreased platelet count	–	–	–	–	–	1 (c4)	3
Treatment delay (*n*)		1	1			2	4
Treatment outcome	Complete response	Stable disease	Progressive disease	Partial response	Progressive disease	Partial response	–

^a^ Pharmacokinetic parameters are presented as geometric mean + interquartile range.

### Treatment and safety outcomes

Table 1 provides an overview of the treatment and outcomes. The first patient was successfully escalated to the 100% dose. No grade III/IV hematological toxicity occurred. Grade I anemia was present at baseline and worsened to grade II during treatment. After four cycles, a complete response was observed. Patient 2 was screened when Patient 1 was treated in the second dose level and, thus, started at the 10% dose level with successful escalation to 100%. At Cycle 2, there was a persistent grade II thrombocytopenia, resulting in a 1‐week treatment delay. During the last cycle, grade III thrombocytopenia grade III occurred at the nadir, which resolved at the end of the cycle. Again, grade I anemia was present at baseline, progressing to grade II during treatment. After four cycles, the patient had stable disease. Patient 3 started at the 66% dose level and was treated at the 100% dose level in Cycles 2–4. Two episodes of thrombocytopenia occurred at the nadir of Cycles 2 and 4, resolving before the next cycle. There was a 1‐week delay due to elevated serum creatinine compared to baseline and the occurrence of a lower airway infection, which was treated with antibiotics, without further affecting treatment. Unfortunately, patient 3 showed progressive disease after four cycles.

In the second cohort, with all patients directly starting at the 100% dose, patients four and five were treated without the occurrence of grade III/IV hematological toxicity. Grade I‐II anemia was seen during treatment, which was present at baseline. Patient four experienced grade I gastrointestinal toxicities, fatigue, and dyspnoea on exertion. This patient was supplemented with calcium and magnesium to treat deficiencies and was shortly admitted for rehydration, reported to be a grade III toxicity resolving after treatment. Patient five experienced grade I gastrointestinal toxicity and grade I alopecia. At the end of four cycles, Patients 4 and 5 showed a partial response and progressive disease, respectively. Before starting, a grade II anemia was already present in patient 6. During treatment, two cycle delays were necessary to recover from grade III pancytopenia. There was one measurement of grade IV decreased neutrophil count in Cycle 4, spontaneously resolving within 1 week. Other AEs were grade I hiccups, hyperpotassemia, cough, and epistaxis. Treatment resulted in a partial response. There were no DLTs that limited pemetrexed treatment or did not resolve without intervention.

### Pharmacokinetics

**Figure 1:**
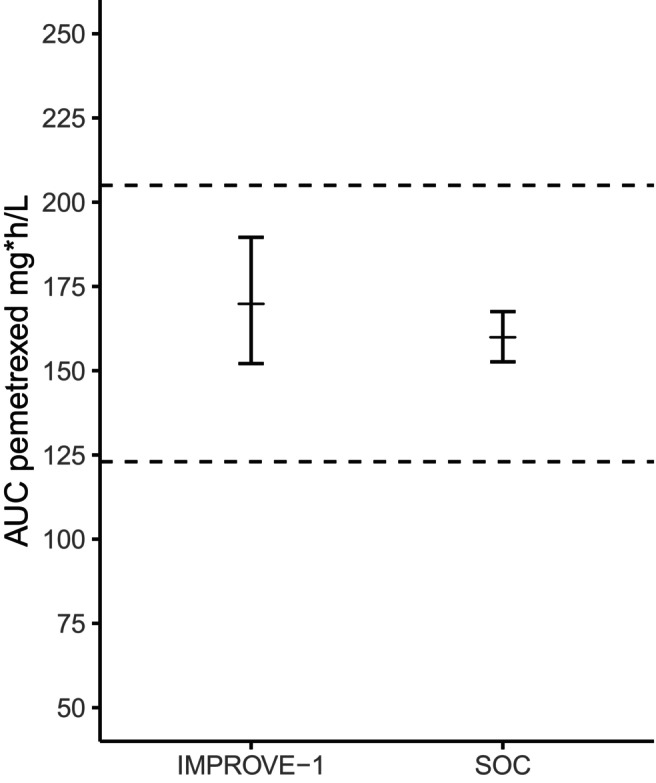
Geometric mean of the area under the concentration–time curve (AUC) plus 90% confidence intervals for patients with impaired renal function from this study (left) and patients with adequate renal function treated conform standard of care (SOC) (pemetrexed 500 mg/m^2^) from the IMPROVE‐II study. The dashed lines indicate the therapeutic range of 164 mg*h/L ± 25%.^9^

**Figure 2:**
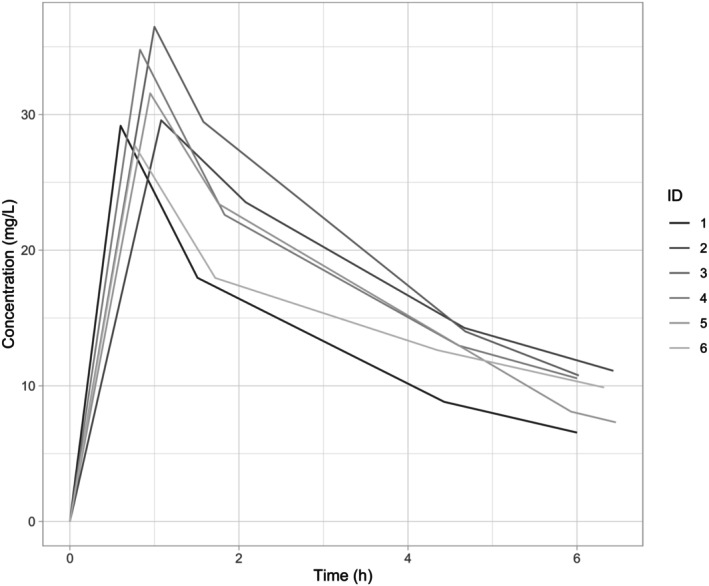
Individual pharmacokinetic curves at 100% dose level.

All patients successfully escalated to full dose and were within the predefined pharmacokinetic target range of 164 mg*/L ± 25% (**Figure** **1**). The geometric mean clearance was 2.39 L/h (interquartile range (IQR) 2.29–2.48) and central volume of distribution (V1) was 6.04 L (IQR5.77–6.58). The geometric mean AUC for patients with renal impairment (*n* = 6) was 170 mg*h/L (90% CI 152–190 mg*h/L). For comparison, the geometric mean AUC with 90% CI for the BSA‐based dosing cohort of the IMPROVE‐II study representing the population with adequate renal function was 171 mg*h/L (90% CI 153–168 mg*h/L), which was within the pre‐established no‐effect boundaries of 123–205 mg*h/L. See **Table** **1** for individual pharmacokinetic results and **Figure** **2** for individual pharmacokinetic curves.

## DISCUSSION

We showed that pemetrexed dosing based on renal function combined with oral folinic acid prophylaxis enables safe treatment and therapeutic exposure in patients with renal impairment, a group that could thus far not be treated with this drug. The renal impairment study of the license holder was halted after one patient (eGFR < 20 mL/min) suffered from fatal toxicities after receiving a 150 mg/m^2^ dose.^3^ In a previous early terminated study, where we dosed pemetrexed based on renal function without folinic acid prophylaxis, three of three patients experienced grade III/IV hematological toxicity at 50% of the target dose.^5^ Phase I–II data show that pemetrexed dosed at 4 mg/m^2^ on five consecutive days and 40 mg/m^2^ once‐weekly resulted in little activity but substantial toxicity. In contrast, 500 mg/m^2^ dosed every 21 days was effective and more tolerable.^17^, ^18^, ^19^ As low, toxic exposure does not seem effective, patients with renal impairment cannot be safely treated without toxicity prophylaxis. We provide the clinical data to support the use of pemetrexed in renal impairment using folinic acid to prevent severe pemetrexed‐related toxicity. This strategy resulted in tolerable treatment with AE patterns and pharmacokinetics equivalent to that in patients with adequate renal function^4^, ^16^ One patient experienced clinically relevant ongoing grade III/IV hematological toxicity leading to treatment delay and was therefore classified as a DLT. Besides dose delay, the DLT resolved without intervention. Two patients experienced grade III/IV hematological toxicity on nadir that resolved before the start of the new treatment cycle. In patients with normal renal function, the reported incidence of grade III/IV neutropenia is also around 15%.^16^ Five patients already had grade I/II anemia at baseline. The KEYNOTE‐189 study found a comparable incidence of hematological toxicity during treatment.^1^ Notably, three of six patients used a proton pump inhibitor (PPI) at baseline for a valid indication. Some evidence suggests that the use of PPI increases the risk of pemetrexed‐related toxicity.^20^ Our pharmacokinetic observation did not confirm a relevant interaction as all patients were within the predefined target. However, as lung cancer patients often have multimorbidity, the use of PPI's cannot be avoided at all times. Preferably, PPIs and NSAIDs should be avoided during the week of pemetrexed administration. Clinicians should be aware of the increased risk for toxicity in case of comedication that interferes with clearance of pemetrexed. Although efficacy was not a primary endpoint in our study, as is common in these studies, we observed signs of clinical efficacy.

The EMA and FDA guidelines state that renal impairment studies need to be performed in case of clinically relevant increase of exposure of a drug is expected in these patients, before applying for market authorization.^7^, ^8^ As a previous study of the license holder failed, this resulted in a negative recommendation for the application of pemetrexed in this patients population in the drug label of pemetrexed. With our study, we show pharmacokinetically equivalent exposure compared with “pivotal efficacy/safety population” is reached in the tested dosing regimen, which complies with the aforementioned guidelines. Geometric mean AUCs were comparable between the patients in the current study compared to a cohort with adequate renal function dosed according to the label.^16^ In our study, we only did pharmacokinetic assessment at the 100% dose level to assess target attainment. The AUC of other dose levels could have provided additional information. However, we assume dose proportional pharmacokinetics, considering the linear pharmacokinetics of pemetrexed in patients with adequate or impaired renal function.^11^ It might be argued that a limitation of the study was the relatively small sample size of six patients. However, the number of a minimum of 6 and a maximum of 12 patients to be included, originates from the traditional 3 + 3 dose escalation design, which is often used in oncology studies and the 90% confidence interval of the pharmacokinetic endpoint was within the equivalence range.^10^ Since our study complies with the regulatory guidelines and inclusion of more patients than required in a study is deemed unethical, six patients were sufficient to develop this treatment strategy in renal impairment.

It should be noted that the eGFR in patients included in our study ranged widely from 26 to 41 ml/min. Nonetheless, it may be argued that the validity of our findings is unknown without the studied range of renal functions. On the other hand, the pemetrexed dose is adjusted for each renal function, and the time of oral folinic acid prophylaxis was based on a worst‐case scenario. Although with caution, we argue that in patients with an eGFR below 26 ml/min, our strategy is likely to result in safe treatment. However, patients and physicians should discuss the risk–benefit ratio in case of severe renal impairment.

For implementation in clinical practice of our strategy, we would like to point out that the timing of starting folinic acid prophylaxis (which should not be mistaken for folic acid, that is also routinely administered during pemetrexed treatment) is of the essence. The first dose of folinic acid in our study was started 24 hours after the infusion, based on a previous analysis that in a typical patient with adequate renal function at the approved dose the systemic pemetrexed concentration decreases below the toxicity threshold.^5^ Although we did not investigate the optimal timing window for folinic acid, from a mechanistical standpoint, it may be argued that starting earlier or later with oral folinic acid prophylaxis results in worse cytotoxic effects or increased toxicity, respectively.

Altogether, we showed that in patients with moderate renal impairment, pemetrexed dose calculated on renal function to reach a target AUC combined with folinic acid prophylaxis resulted in safe treatment and systemic exposure equivalent to the exposure reached with the approved dose in patients with adequate renal function. Although the data for patients with severe renal impairment are scarce, we postulate that our strategy can be applied for these patients. Real‐world data should be gathered to confirm this. The provided level of evidence complies with regulatory guidelines; thus, our pemetrexed treatment strategy for patients with renal impairment can be implemented in routine care. This strategy unlocks the potential of chemoimmunotherapy in NSCLC patients that were, thus far, withheld from effective treatment.

## FUNDING

This research was funded by ZonMW, project number: 848016010.

## CONFLICTS OF INTEREST

*BP*: Research funding: Amgen, Treatmeds. Advisory boards: Bristol‐Myers Squibb, Janssen, Pfizer, Takeda, Merck. Advisory/Speaker fees: AstraZeneca, Janssen, Pfizer. *All fees paid to the institution/department, no personal fees*. Other: Committee member Off‐label Oncological Medication (CIE‐OOM). Guideline committee: Patient counseling for genetic tumordiagnostics. Board Member Netherlands Respiratory Society. CS: Research funding: AstraZanaeca, NVALT studies, ICON Clinical Research/Arcus biosciences, VitroScan Leiden. Advisory: BMS. Other: Lilly. *AD:* Institutional fees from Roche, Eli Lilly, Boehringer Ingelheim, AstraZeneca, Janssen, Chiezi, Amgen, Pfizer, Bayer, Takeda, Pharmamar, Sanofi, and Daiichi; *RM:* Research funding paid to the institute from Astellas, Bayer, Boehringer‐Ingelheim, Cristal Therapeutics, Novartis, Pamgene, Pfizer, Roche, Sanofi, and Servier. *BvV:* Advisory/Speaker fees: Astra‐Zeneca, BMS, Novartis, Lilly, Roche. *LH:* Research funding Roche Genentech, AstraZeneca, Boehringer Ingelheim, Takeda, Merck, Pfizer, Novartis, Gilead. Speaker educationals/webinars: AstraZeneca, Bayer, Lilly, MSD, high5oncology, Takeda, Janssen, GSK, Sanofi, Pfizer (Inst), Medtalks, Benecke, VJOncology, Medimix (self). Advisory boards: Amgen, Boehringer Ingelheim, Lilly, Novartis, Pfizer, Takeda, Merck, Janssen, MSD, Anheart, Bayer, AZ. Member guideline committees: Dutch guidelines on NSCLC, brain metastases, and leptomeningeal metastases (self), ESMO guidelines on metastatic NSCLC and SCLC (non‐financial). Other (non‐financial): secretary NVALT studies foundation, subchair EORTC metastatic NSCLC systemic therapy, vice‐chair scientific committee Dutch Thoracic Group. *DD:* Advisory/Speaker fees: Amgen, BMS, MSD, Novartis, Roche. *MvdH:* Research funding: AstraZeneca, BMS, Janssen, Treatmeds, Merck, MSD, Novartis, Pfizer, Roche, Roche diagnostics. Advisory/Speaker fees: Abbvie, AstraZeneca, BMS, Lilly, MSD, Novartis, Pfizer, Roche. Other: SON‐NVALT, NVALTstudies, Longkankernet. *RtH:* Research funding: Amgen. All other authors declared no competing interests for this work.

## AUTHOR CONTRIBUTIONS

N.d.R., LS.O, M.P.K, B.P., B.B., B.v.V., C.M.J.S., B.vd.B, L.E.L.H., S.C., AM.C.D., D.W.D., R.H.J.M., A.D.R.H., H.J.D., M.J.D., M.M.vd.H., J.A.B., and R.t.H. wrote the manuscript. N.d.R., LS.O., B.P., B.B., B.v.V., L.E.L.H., S.C., AM.C.D., R.H.J.M., A.D.R.H., H.J.D., M.M.vd.H, J.A.B., and R.t.H. designed the research. N.d.R., LS.O., M.P.K., B.P., B.B., B.v.V., C.M.J.S., B.vd.B., L.E.L.H., D.W.D., M.M.vd.H., J.A.B., and R.t.H. performed the research. N.d.R., LS.O., and R.t.H. analyzed the data.

## TRIAL REGISTRATION

Clinicaltrials.gov: NCT03656549.
